# Understanding the Complexity of Temperature Dynamics in Xinjiang, China, from Multitemporal Scale and Spatial Perspectives

**DOI:** 10.1155/2013/259248

**Published:** 2013-06-13

**Authors:** Jianhua Xu, Yaning Chen, Weihong Li, Zuhan Liu, Chunmeng Wei, Jie Tang

**Affiliations:** ^1^The Research Center for East-West Cooperation in China, The Key Laboratory of Geographic Information Science, Ministry of Education of PRC, East China Normal University, Shanghai 200241, China; ^2^State Key Laboratory of Desert and Oasis Ecology, Xinjiang Institute of Ecology and Geography, Chinese Academy of Sciences, Urumqi 830011, China

## Abstract

Based on the observed data from 51 meteorological stations during the period from 1958 to 2012 in Xinjiang, China, we investigated the complexity of temperature dynamics from the temporal and spatial perspectives by using a comprehensive approach including the correlation dimension (CD), classical statistics, and geostatistics. The main conclusions are as follows (1) The integer CD values indicate that the temperature dynamics are a complex and chaotic system, which is sensitive to the initial conditions. (2) The complexity of temperature dynamics decreases along with the increase of temporal scale. To describe the temperature dynamics, at least 3 independent variables are needed at daily scale, whereas at least 2 independent variables are needed at monthly, seasonal, and annual scales. (3) The spatial patterns of CD values at different temporal scales indicate that the complex temperature dynamics are derived from the complex landform.

## 1. Introduction

The Earth's climate system is a complex, interconnected system formed by the atmosphere, the oceans, and other bodies of water, land surface, snow, and ice cover together with all living organisms and linked by flows of energy and matter. To discover the complexity of climate change process, many concepts and methods, such as entropy, fractal, nonlinearity, chaos, wavelet, and artificial neural network, have been used by scholars [1–6]. The climate models used in the research on climate change have become more complex and are today believed to be able to provide fairly reliable predictions of future temperature ranges and climate developments. However, the models still do not cover a complete set of possible mechanisms and they include considerable uncertainties. Specifically, applying global scale simulation results to interpreting and predicting regional situations is challenging, and in fact its applicability is questionable [7].

Studies have suggested that the climatic process is a chaotic dynamic system, with nonlinearity as its basic characteristic; nevertheless, there are still many open questions on the complex system [3, 5, 8, 9] such as how to understand the complexity of spatial and temporal scales of the regional climatic system; the question has no satisfactory answer.

In the last 20 years, many studies have been conducted to evaluate climatic change in the arid and semiarid regions in northwestern China [10–14]. Some studies reached a conclusion that there was a visible climatic transition in the past half-century [15–18]. This transition was characterized by a temperature increase trend. However, the conclusion brought a question whether the increase trend is a regional response to global warming or merely a rising stage in the periodic dynamic process [19].

In order to understand the complexity of climatic dynamics in Xinjiang, China, based on observed data at 51 meteorological stations during the period from 1958 to 2012, this study investigated the temperature dynamics from multiple temporal scale and spatial perspectives by using a comprehensive approach including the correlation dimension (CD), classical statistics, and geostatistics.

## 2. Study Area and Data

### 2.1. Study Area

**Figure 1:**
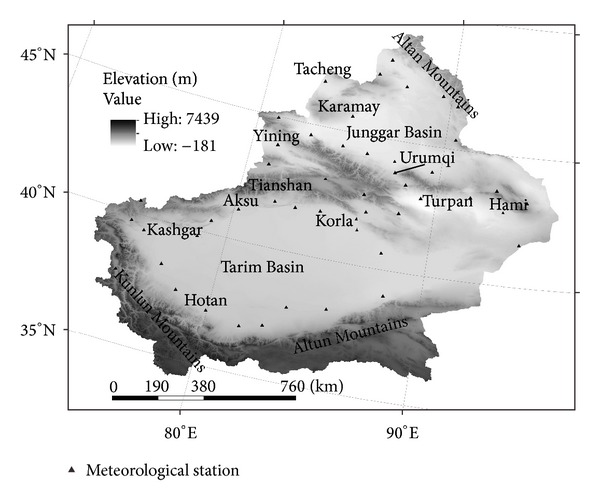
Elevation and locations of meteorological stations in the study area.

Located in the northwest of China, Xinjiang is a typical semiarid and arid area. It extends between 73°40′–96°23′E and 34°25′–48°10′N and covers an area of 166.04 × 10^4^ km^2^ (Figure 1). There are three mountain ranges in Xinjiang. From south to north, they are Kunlun, Tianshan, and Altay mountains. With their high elevations, these mountains block atmospheric circulations and create two vast desert basins in their rain-shadows between the mountains, that is, the Tarim Basin in the south and the Junggar Basin in the north. The Tianshan Mountain in the middle divides Xinjiang into the northern and southern parts. Northern Xinjiang has a continental arid and semiarid climate, with a mean temperature of −13°C in winter and 22.2°C in summer. Southern Xinjiang has a continental dry climate, with a winter mean temperature of −5.7°C and a summer mean temperature of 24.4°C. Annual precipitation is about 210 mm in northern Xinjiang while southern Xinjiang has less than 100 mm. Because of the dry climate, evaporation in Xinjiang is very strong with a mean annual pan evaporation between 1000 and 4500 mm, which is 500–1000 mm higher than other places at the same latitude in China [11, 20].

### 2.2. Data

To ensure consistency and the longest continuous observation, data from 51 ground-based meteorological stations of the China Meteorological Administration (CMA) were used in this study. In order to understand the complexity of climate process from a multitemporal scale perspective, the daily, monthly, seasonal, and annual air temperature data from 1958 to 2012 were used for computation and analysis.

## 3. Methods

In order to understand the complexity of temperature dynamics in Xinjiang, China, this paper conducted an integrative approach combining the correlation dimension (CD), classical statistics, and geostatistics method. Firstly, the CD value was computed to show the chaotic and fractal characteristics of temperature dynamics at different temporal scales. Secondly, correlations between the CD value with geographical location and elevation was showed by the correlation analysis and stepwise regression. Finally, the variogram and cokriging methods were used to reveal the spatial pattern of the CD values.

### 3.1. Correlation Dimension

The correlation dimension (CD) is usually applied to analyze a time series and determine if it exhibits a chaotic dynamic characteristic [21, 22]. Consider *x*(*t*), the time series of annual runoff, and suppose that it is generated by a nonlinear dynamic system with *m* degrees of freedom. To restore the dynamic characteristic of the original system, it is necessary to construct an appropriate series of state vectors, *X* ^(*m*)^(*t*), with delay coordinates in the *m*-dimensional phase space according to the basic ideas initiated by Grassberger and Procaccia [23]:

(1) $$\begin{array}{c} X^{\left(m\right)}\left(t\right)=\left\{x\left(t\right),x\left(t+\tau \right),\ldots ,x\left(t+\left(m-1\right)\tau \right)\right\}, \end{array}$$

where *m* is the embedding dimension and *τ* is an appropriate time delay.

The trajectory in the phase space is defined as a sequence of *m*-dimensional vectors. If the dynamics of the system can be reduced to a set of deterministic laws, the trajectories of the system converge toward a subset of the phase space, which is called an “attractor.” Many natural systems do not conform with time to a cyclic trajectory. Some nonlinear dissipative dynamic systems tend to shift toward the attractors for which the motion is chaotic, that is, not periodic and unpredictable over long times. The attractors of such systems are called strange attractors. For the set of points on the attractor, using the G-P method [23], the correlation-integrals are defined to distinguish between stochastic and chaotic behaviors.

The correlation-integrals can be defined as follows:

(2) $$\begin{array}{c} C\left(r\right)=\frac{1}{N_{R}^{2}}\sum_{j=1}^{N_{R}}\;\sum_{i=1}^{N_{R}}\Theta \left(r-\left|X_{i}-X_{j}\right|\right), \end{array}$$

where *r* is the surveyor's rod for distance, *N*_*R*_ is the number of reference points taken from *N*, and *N* is the number of points, *X* ^(*m*)^(*t*). The relationship between *N* and *N*_*R*_ is *N*_*R*_ = *N* − (*m* − 1)*τ*. Θ(*x*) is the Heaviside function, which is defined as

(3) $$\begin{array}{c} \Theta \left(x\right)=\{\begin{array}{cc} 0 & x\leq 0\\ 1 & x>0. \end{array} \end{array}$$

The expression counts the number of points in the dataset that are closer than the radius, *r*, within a hypersphere of the radius, *r*, and then divides this value by the square of the total number of points (because of normalization). As *r* → 0, the correlation exponent, *d*, is defined as

(4) $$\begin{array}{c} C\left(r\right)\propto r^{d}. \end{array}$$

**Figure 2:**
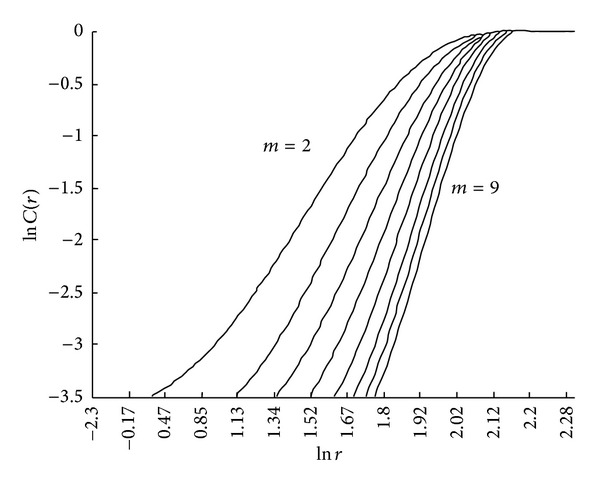
A plot of ln⁡*C*(*r*) versus ln⁡(*r*).

It is apparent that the correlation exponent, *d*, is given by the slope coefficient of ln⁡*C*(*r*) versus ln⁡*r*. According to (ln⁡*r*, ln⁡*C*(*r*)), *d* can be obtained by the least squares method (LSM) using a log-log grid (as shown in Figure 2).

**Figure 3:**
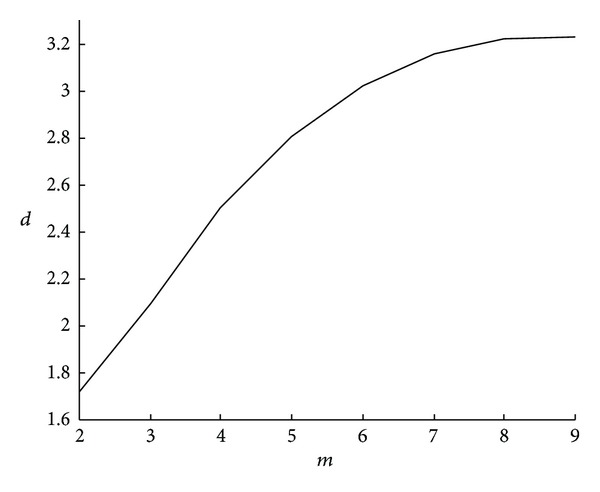
The correlation exponent (*d*) versus embedding dimension (*m*).

To detect the chaotic behavior of the system, the correlation exponent has to be plotted as a function of the embedding dimension (as shown in Figure 3). 

If the system is purely random (e.g., white noise), the correlation exponent increases as the embedding dimension increases, without reaching the saturation value. If there are deterministic dynamics in the system, the correlation exponent reaches the saturation value, which means that it remains approximately constant as the embedding dimension increases. The saturated correlation exponent is called the correlation dimension (CD) of the attractor. The CD belongs to the invariants of the motion on the attractor. It is generally assumed that the CD equals the number of degrees of freedom of the system, and higher embedding dimensions are therefore redundant. For example, to describe the position of the point on the plane (two-dimensional system), the third dimension is not necessary because it is redundant. In addition, the CD value is often fractal and represented as a nonintegral dimension, which is typical for chaotic dynamical systems that are very sensitive to initial conditions.

The CD value provides the information regarding the dimension of the phase-space required for embedding the attractor. It is important for determining the number of dimensions necessary to embed the attractor and the number of variables present in the evolution of the process.

We used the previous correlation dimension method to analyze the chaotic and fractal characteristics for the temperature dynamics in this study.

### 3.2. Correlation Analysis and Stepwise Regression

Correlation and regression analyses are the two commonly useful methods in various disciplines of geography [24], which were used to check the correlations between the CD value with geographical location and elevation in this study.

The correlation analysis is one of the most useful classical statistics, which is a statistical measurement of the correlationship between two variables. Possible correlations range from +1 to –1. A zero correlation indicates that there is no relationship between the variables. A negative correlation indicates that as one variable goes up, the other goes down. A positive correlation indicates that both variables move in the same direction together.

For the two variables, *x* and *y*, the correlation coefficient is calculated as

(5) $$\begin{array}{c} r_{xy}=\frac{\sum_{i=1}^{n}\left(x_{i}-\overset{-}{x}\right)\left(y_{i}-\overset{-}{y}\right)}{\sqrt{\sum_{i=1}^{n}{\left(x_{i}-\overset{-}{x}\right)}^{2}}\sqrt{\sum_{i=1}^{n}{\left(y_{i}-\overset{-}{y}\right)}^{2}}}, \end{array}$$

where *n* is the sample number; *x*_*i*_ represents the value of *x* for the sample *i*; *y*_*i*_ represents the value of *y* for the sample *i*; $\overset{-}{x}$ is the mean for all *x*_*i*_; $\overset{-}{y}$ is the mean for all *y*_*i*_. Commonly, testing the significance of the correlation coefficient employs the *t* distribution.

Stepwise regression can be achieved either by trying out one independent variable at a time and including it in the regression model if it is statistically significant, or by including all potential independent variables in the model and eliminating those that are not statistically significant, or by a combination of both methods. The multiple linear regression equation (MLRE) is as follows:

(6) $$\begin{array}{c} Y=a_{0}+a_{1}X_{1}+a_{2}X_{2}+\cdots +a_{k}X_{k}, \end{array}$$

where *Y* is dependent variable and *a*_*i*_ is the coefficient of the independent variables *X*_*i*_ (*i* = 1,2,…, *k*). In this study, the dependent variable is the CD value and the independent variables are elevation, latitude, and longitude.

### 3.3. Geostatistics

Studies have shown that the parameters of temperature dynamics are typical regionalized variables, which are structural as well as stochastic [25, 26]. So its spatial variability can be analyzed by the geostatistics method [27, 28].

#### 3.3.1. The Variogram

The regionalized variable is regarded as the value of a variable at a location *x* as a realization of a stochastic *Z*(*x*). This stochastic is assumed to be intrinsically stationary. The first is that the expected value of the stochastic, *E* [*Z*(*x*)], is constant for all *x*. Secondly, the variance of the differences between the values of the variable at two different locations depends only on the lag vector separating the two locations and not on the absolute locations. In general, this variance may be a function of both the direction and length of the lag vector. If the regionalized variable is isotropic, the variogram is purely a function of the length of the vector which we denote by *h*. Thus the relationship between values from different locations is described by the variogram as follows [27, 28]:

(7) $$\begin{array}{c} \gamma \left(h\right)=\frac{1}{2}E\left[{\left(Z\left(x\right)-Z\left(x+h\right)\right)}^{2}\right]. \end{array}$$

The variogram is estimated from variable values observed at sampled points, *x*_*s*_, *s* = 1,…, *n*. The method of estimator is the average of squared differences between observations separated by distance *h* as follows:

(8) $$\begin{array}{c} \gamma \left(h\right)=\frac{1}{2N\left(h\right)}\sum_{i=1}^{N(h)}{\left[Z\left(x_{i}\right)-Z\left(x_{i}+h\right)\right]}^{2}, \end{array}$$

where *Z*(*x*_*i*_) indicates the magnitude of regionalized variable and *N*(*h*) is the total number of pairs of attributes that are separated by a distance *h*.

#### 3.3.2. Kriging and Cokriging Methods

Based on the variogram, Kriging and cokriging can be used to estimate the values of regionalized variable at unsampled locations [29, 30].

Ordinary Kriging can mathematically be defined as given in the following:

(9) $$\begin{array}{c} Z_{X}^{*}=\sum_{i=1}^{n}\lambda_{i}Z\left(X_{i}\right), \end{array}$$

where *Z*_X_* is the estimated value and *λ*_*i*_ is the corresponding weight of each observation *Z*(*X*_*i*_) on the estimation. These weights are calculated to ensure that the estimator is unbiased and the estimation variance is a minimum. The nonbias condition requires that

(10) $$\begin{array}{c} \sum_{i=1}^{n}\lambda_{i}\gamma \left(X_{i},X_{j}\right)-\mu =\gamma \left(X_{i},X^{*}\right),\\ \sum_{i=1}^{n}\lambda_{i}=1, \end{array}$$

where *γ*(*X*_*i*_, *X*_*j*_) is the variogram between sampled point *i* and point *j*, *γ*(*X*_*i*_, X*) is the variogram between sampled point and estimated point, and *μ* is the Lagrange multiplier of minimum condition.

The general form of cokriging equations is

(11) $$\begin{array}{c} \sum_{l=1}^{v}\;\sum_{i=1}^{n_{l}}\lambda_{il}\gamma_{lv}\left(X_{i},X_{j}\right)-\mu_{v}=\gamma_{uv}\left(X_{j},X^{*}\right),\\ \sum_{i=1}^{n_{l}}\lambda_{il}=\{\begin{array}{cc} 1, & l=u\\ 0, & l\neq u, \end{array} \end{array}$$

where *u* and *v* are the primary and covariate (secondary) variables, respectively. In the cokriging method, the *u* and *v* are cross-correlated and the covariate contributes to the estimation of the primary variable. Generally, measuring the covariate is simpler than measuring the primary variable. For cokriging analysis, the cross variogram (or cross-variogram) should be determined in prior. Provided that there are points where both *u* and *v* have been measured, the cross-variogram is estimated by

(12) γuv(h)=12N(h)∑i=1N(h)[Zu(Xi)−Zu(Xi+h)]        ×[Zv(Xi)−Zv(Xi+h)].

## 4. Results and Discussion

### 4.1. The Chaotic Dynamic Process

Based on the meteorological data, we analyzed the chaotic dynamics with fractal characteristic for the temperature dynamics by using the G-P method [23].

**Figure 4:**
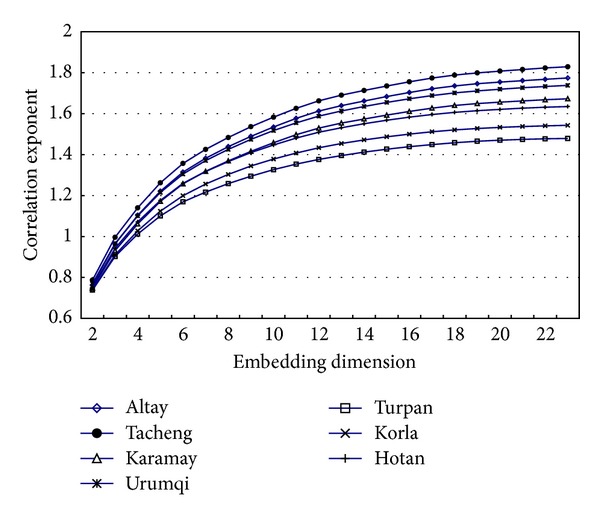
The plots of correlation exponent (*d*) versus embedding dimension (*m*) for the time series of monthly data from the selected 7 meteorological stations.

Firstly, we randomly selected the time series of monthly data from 7 meteorological stations (i.e., Altay, Tacheng, Karamay, Urumqi, Turpan, Korla, and Hotan station) for a pilot study. The plots of correlation exponent (*d*) versus embedding dimension (*m*) were drawn as Figure 4.

Each plot in Figure 4 showed the gradual saturation process of the correlation exponent. It is evident that the correlation exponent increases with embedding dimension, *m*, and a saturated correlation exponent, the correlation dimension of attractor, that is, CD, was obtained when *m* ≥ 20.

Then, we repeated the previous process for the time series of daily, monthly, seasonal, and annual data in each meteorological station, and the results revealed that the correlation exponent reached the saturation value, which demonstrated that there is correlation dimension of the attractor in the temperature process at the each temporal scale (i.e., daily, monthly, seasonal, and annual scales).

Table 1 showed the correlation dimensions, that is, CD values, at daily, monthly, seasonal, and annual scales for 51 meteorological stations.

Because none of the CD values in Table 1 is integer, this indicated that the temperature process at each temporal scale is chaotic dynamic system with a fractal characteristic and is sensitive to the initial conditions.

### 4.2. The Complexity of Temporal Scale

The last row in Table 1 showed the mean of CD values for every meteorological station at daily, monthly, seasonal, and annual scales.

The order of the MCD (2.5353 > 1.6397 > 1.4156 > 1.2995) reveals the complex order of the temperature dynamics at daily, monthly, seasonal, and annual scales; that is, the complexity of temperature dynamics decreases along with the increase of temporal scale. We think that the results accord with the facts, because the daily data series contains more details, and then followed by monthly data series, seasonal data series, and annual daily data series, respectively. Thus, we conclude that the temperature process at a smaller temporal scale is more complex than that at a larger temporal scale.

The MCD values also provided the information about the dimension of the phase-space required for embedding the attractor. Because all the CD values at daily scale are above 2, at least 3 independent variables are needed at to describe the dynamics of temperature process at daily scale. The same reason tells us that at least 2 independent variables are needed to describe the dynamics of temperature process at monthly, seasonal, and annual scales.

### 4.3. The Effect of Geographical Location and Elevation

Table 1 showed that the CD values at different sites (the sites of meteorological station) are different at a same temporal scale (i.e., daily, monthly, seasonal, and annual scales). Maybe their spatial patterns are affected by the geographical location and elevation.

To reveal the correlation of the CD value with geographical location and elevation, we computed the correlation coefficients as in Table 2.

Table 2 showed that on the daily scale, the CD value positively correlates with elevation at the significant level of 0.05, whereas on the monthly scale, the CD value positively correlates both with elevation and latitude at the significant levels of 0.05 and 0.01, respectively. To verify the correlation, we used the stepwise regression analysis method to fit the multiple linear regression equations (MLREs) between the CD value with geographical location and elevation at daily and monthly scales, which are as in Table 3.

Table 3 told us that on the daily and monthly scales, the CD values are well explained by the geographical location and elevation at the significant levels of 0.006 and 0.000. The MLREs in Table 3 indicate that the site with higher elevation and latitude has a higher CD value. That is to say, the temperature dynamics at the site with higher elevation and latitude are of much higher complexity.

Though the MLREs in Table 3 well explained the relation between CD value with geographical location and elevation at daily and monthly scales, the CD value has no significant correlation with elevation, latitude, and longitude at the seasonal and annual scales. What is the reason for this? 

Actually, beside the structural factor such as atmospheric circulation, the local temperature dynamics are also affected by the location, elevation, and other stochastic factors. Therefore, the CD value of temperature dynamic is a typical regionalized variable and its spatial pattern should be described by the variogram.

By using the aforementioned method for computing variogram, we fitted two variograms to describe the spatial variability of CD value at the seasonal and annual scales.

At seasonal scale, the spatial variability of CD value was well described by the variogram of Gaussian model as follows:

(13) $$\begin{array}{c} \gamma \left(h\right)=\{\begin{array}{cc} 0 & h=0\\ 0.0013049+0.00013166\left(1-e^{-h^{2}/6.93^{2}}\right) & h>0, \end{array} \end{array}$$

where *γ*(*h*) is the value of variogram, and *h* is distance. The mean error and average standard error for model (13) are −0.0008275988 and 0.1726933, respectively.

At annual scale, the variogram of Gaussian model well described the spatial variability of CD value as follows:

(14) $$\begin{array}{c} \gamma \left(h\right)=\{\begin{array}{cc} 0 & h=0\\ 0.025911+0.0000042869\left(1-e^{-h^{2}/6.99^{2}}\right) & h>0, \end{array} \end{array}$$

where *γ*(*h*) and *h* have the same meaning as in formula (13). The mean error and average standard error for model (14) are 0.0001671542 and 0.1709583, respectively.

Based on the previous models of variogram (13) and (14), choosing elevation and latitude as the two covariate variables, we used the aforementioned cokriging method to compute the interpolating of CD values at seasonal and annual scales.

**Figure 5:**
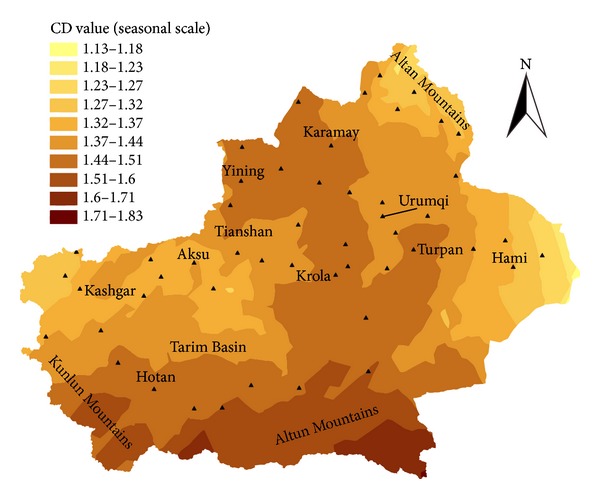
The spatial pattern of CD values at seasonal scale.

Figure 5 presented the spatial pattern of CD values at seasonal scale, which showed that all the CD values are between 1.13 and 1.83. The higher values mainly distribute in the Tianshan, Kunlun, and Altun Mountains, which indicates that the temperature dynamics in these mountain areas are more complicated than other areas. The lower values mainly distribute in the Tarim Basin and the Hami Basin, which indicates that the complexity of the temperature dynamics in these basin areas is comparatively lower than other areas.

**Figure 6:**
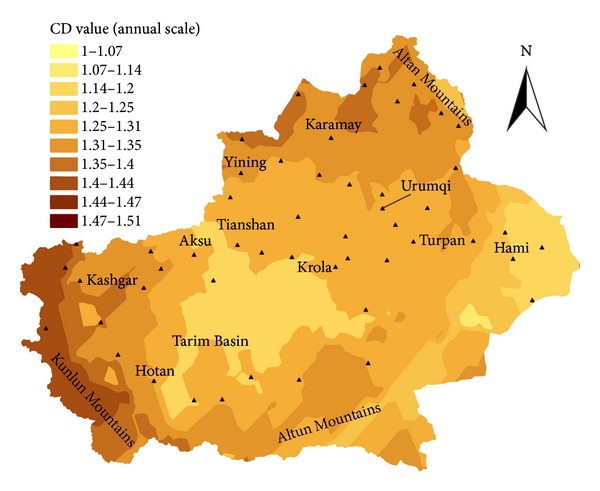
The spatial pattern of CD values at annual scale.

Figure 6 presented the spatial pattern of CD values at seasonal scale, which showed that all the CD values are between 1 and 1.51. Comparing it with Figure 5, the pattern of spatial distribution is a little different. The higher values mainly distribute in the Junggar Basin and part of the Altan, Kunlun, and Altun Mountains, whereas the lower values mainly distribute in the Tarim Basin, the Turpan Basin, and the Hami Basin.

Summarizing the results of Section 4.3, we came to the results at seasonal and annual scales as that the higher CD values mainly distribute on complex landform such as mountain areas, whereas the lower CD values mainly distribute on the comparative flat landform such as basin area. The results indicate that the complex temperature dynamics are derived from the complex landform.

## 5. Conclusion

Summarizing the previous results, we elicited the conclusions as follows.

1. The integer CD values indicate that the temperature dynamics are a complex and chaotic system, which is sensitive to the initial conditions.
2. The order of the MCD (2.5353 > 1.6397 > 1.4156 > 1.2995) reveals the complex order of the temperature dynamics at daily, monthly, seasonal, and annual scales, that is, the complexity of temperature dynamics decreases along with the increase of temporal scale. To describe the temperature dynamics, at least 3 independent variables are needed at daily scale, whereas at least 2 independent variables are needed at monthly, seasonal, and annual scales.
3. The MLREs at the daily and monthly scales show that the site with higher elevation and latitude has a higher CD value, which indicates that the temperature dynamics at the site with higher elevation and latitude are of much higher complexity.
4. The results of the interpolating from cokriging method based on the variogram at seasonal and annual scales show that the higher CD values mainly distribute on complex landform such as mountain area, while the lower CD values mainly distribute on the comparative flat landform such as basin area. The results indicate that the complex temperature dynamics come from the complex landform.

## Figures and Tables

**Table 1:** CD values at daily, monthly, seasonal, and annual scales for 51 meteorological stations.

Station	Temporal scale			
	Annual	Seasonal	Monthly	Daily
Habahe	1.3399	1.2538	1.7895	2.6373
Jeminay	1.4376	1.2197	1.7828	2.7597
Fuhai	1.2639	1.2245	1.6791	2.1925
Fuyun	1.3165	1.1562	1.7068	2.5836
Tacheng	1.4476	1.6813	1.8233	2.6653
Qinghe	1.2545	1.3797	1.7050	2.5283
Karamay	1.2587	1.4392	1.6682	2.4932
Beitashan	1.3605	1.4794	1.6964	2.7709
Wenquan	1.0238	1.6663	1.6578	2.5968
Jinghe	1.4741	1.4956	1.6771	2.4513
Wusu	1.4652	1.3343	1.6392	2.5162
Shihezi	1.2473	1.3688	1.6659	2.5461
Caijiahu	1.2623	1.3639	1.5986	2.4569
Yining	1.4580	1.4899	1.7926	2.6267
Zhaosu	1.4339	1.2854	1.7921	2.7418
Urumqi	1.4037	1.5881	1.7196	2.6578
Balguntay	1.0261	1.5104	1.6349	2.6119
Dabancheng	1.2890	1.5776	1.6765	2.5897
Shisanjianfang	1.2165	1.3803	1.6418	2.5630
Kumishi	1.0826	1.2733	1.5108	2.4163
Bayinbuluke	1.3018	1.8288	1.7219	2.5775
Yanqi	1.3682	1.4965	1.5582	2.4040
Turpan	1.3872	1.3478	1.4744	2.4458
Akzo	1.3614	1.3917	1.5661	2.4937
Baicheng	1.1443	1.2759	1.5931	2.4353
Luntai	1.0844	1.3784	1.5369	2.3993
Kuche	1.1113	1.3517	1.5945	2.5045
Torugart	1.4475	1.2478	1.7755	2.6989
Wuqia	1.2710	1.2497	1.7606	2.6237
Kashgar	1.4516	1.2459	1.6445	2.4876
Bachu	1.0044	1.2648	1.5567	2.4941
Kalpin	1.2194	1.3801	1.5522	2.4987
Tieganlike	1.3480	1.5565	1.4824	2.5297
Ruoqiang	1.3782	1.6542	1.4764	2.5498
Tashkuergan	1.4328	1.5879	1.8265	2.6284
Shache	1.4771	1.3357	1.5625	2.5330
Pishan	1.3998	1.6722	1.6570	2.5574
Khotan	1.3830	1.4586	1.6318	2.5564
Minfeng	1.1925	1.2186	1.5654	2.5446
Qiemo	1.4022	1.7546	1.5209	2.0532
Yutian	1.4214	1.5872	1.5632	2.5299
Barkol	1.0545	1.5801	1.6959	2.6697
Hami	1.4728	1.1299	1.5723	2.5422
Hongliuhe	1.0456	1.2998	1.6512	2.1780
Altay	1.5126	1.6475	1.7613	2.6203
Qitai	1.4519	1.3791	1.6876	2.5112
Korla	1.3495	1.2753	1.5945	2.5238
Aheqi	1.4504	1.5167	1.6792	2.6041
Alar	1.0457	1.3586	1.4358	2.4821
Andehe	1.2264	1.3956	1.4202	2.6490
Yiwu	1.0170	1.1602	1.6505	2.5685

MCD	1.2995	1.4156	1.6397	2.5353

Note: MCD is the mean of correlation dimensions for all meteorological stations.

**Table 2:** The correlation coefficients between CD values with geographical location and elevation.

	CD			
	Annual	Seasonal	Monthly	Daily
Elevation	−0.0590	0.1145	0.2927*	0.2854*
Latitude	0.0287	−0.1101	0.5002**	0.1786
Longitude	−0.2242	−0.0824	−0.0999	−0.1589

Notes: **correlated at significance level of 0.01; *correlated at significance level of 0.05.

**Table 3:** MLREs between the CD values with geographical location and elevation at daily and monthly scales.

Temporal scale	Regression equation	*F*	Significant level
Daily	CD = 0.008919*x*_1_ + 0.016*x*_2_ + 1.752	5.667	0.006
Monthly	CD = 0.009517*x*_1_ + 0.029776*x*_2_ + 0.450	30.722	0.000

Note: CD is the value of correlation dimension; *x*_1_ is elevation (10^2^ m); *x*_2_ is latitude (°C).
